# Masses stromales gastro-intestinales multiples du grêle découvertes dans un contexte de péritonite

**DOI:** 10.11604/pamj.2019.33.102.17240

**Published:** 2019-06-11

**Authors:** Dondo Mara, Moussa Sylla, Seydou Ly, Ismaël Dandakoye Soumana, Badr Alami, Mustapha Maâroufi, Khalid Maazaz, Youssef Alaoui Lamrani

**Affiliations:** 1 Service de Radiologie, CHU Hassan II Fès, Maroc; 2 Service de Chirurgie Viscérale, CHU Hassan II Fès, Maroc

**Keywords:** GIST du grêle, péritonite, tomodensitométrie, Gastrointestinal stromal tumors (GIST) in the small bowel, peritonitis, CT scan

## Abstract

Nous rapportons une observation d'un cas de tumeur stromale gastro-intestinale à localisation multiple au niveau du grêle découverte dans un contexte de péritonite. La particularité de ce cas tient à la découverte en per-opératoire de masses tumorales multifocales grêliques, évoquant à la tomodensitométrie en post opératoire des tumeurs stromales gastro-intestinales. Ce tableau clinique de péritonite ne permettait en aucun cas d'évoquer une origine tumorale.

## Abstract (en)

We here report a case of multiple gastrointestinal stromal tumors (GIST) in the small bowel detected in a patient with peritonitis. The peculiarity of this case study is the intraoperative detection of multifocal small bowel tumor masses, suggesting gastrointestinal stromal tumors on postoperative CT scan. Tumor couldn't be suspected clinically on the basis of peritonitis.

## Introduction

C'est en 1998, après la découverte de mutations du gène de fonction dans le proto-oncogène c-KIT, que les tumeurs stromales gastro-intestinales se sont distinguées des autres sous-types histopathologiques des tumeurs mésenchymateuses [1, 2]. Les tumeurs stromales gastro-intestinales (GIST) représentent les néoplasmes mésenchymateux les plus courants du tractus gastro-intestinal. Avec une incidence annuelle de 11-14 pour 106, elles forment 0,1% -3,0% des tumeurs malignes gastro-intestinales [3, 4]. Seulement 70% des patients atteints de GIST sont symptomatiques. Alors que 20% sont asymptomatiques et sont détectées accidentellement, et 10% des lésions ne sont détectées qu'à l'autopsie. Les symptômes ne sont pas spécifiques à la maladie, ils sont plus liés au site de la tumeur [4-6]. Le scanner avec injection du produit de contraste est la modalité diagnostique de choix; il permet de caractériser la lésion, évaluer son étendue et rechercher la présence ou non de métastases lors du bilan initial. La tomodensitométrie est également utilisée pour évaluer la réponse à la thérapie et assurer une surveillance post thérapeutique adéquate [7-9]. Les tumeurs stromales multifocales de siège grêlique découvertes dans un contexte de péritonite sont rarement rapportées par la littérature. Nous rapportons ici l'observation d'un sujet de sexe masculin âgé de 55 ans présentant une tumeur stromale multifocale découverte lors d'une intervention chirurgicale pour péritonite.

## Patient et observation

**Figure 1:**
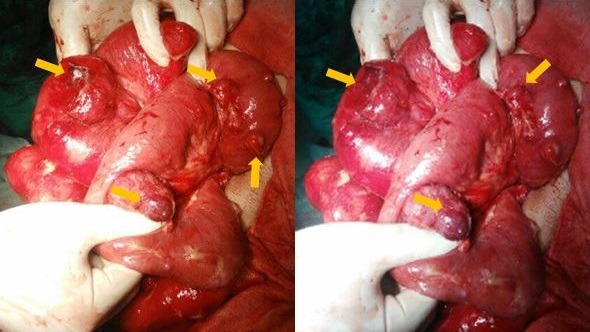
Image per opératoire: masses tumorales au dépend du grêle

**Figure 2:**
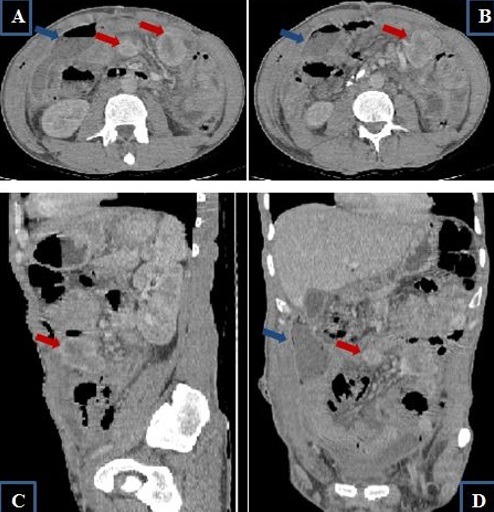
Image scannographique en coupe axiale (A et B) sagittale (C) et coronale (D) avec injection du PDC au temps portal: volumineuse collection du flanc droit à contenu hydro-aérique et à paroi rehaussée après contraste: quelques masses intéressant le jéjunum avec un rehaussement périphérique et hétérogène après contraste avec une liquéfaction centrale (nécrose)

Il s'agit d'un patient âgé de 55 ans admis dans notre structure pour complication post opératoire à j9 d'une péritonite aiguë. En effet l'examen clinique initial avait mis en évidence un tableau d'abdomen aigu sans orientation étiologique qui nécessitait une prise en charge chirurgicale d'urgence. Cependant une échographie a été réalisée en urgence et a mis en évidence la présence de multiples masses intra péritonéales dont la plus grande était hétérogène et mesurait approximativement 3cm de grand axe associée à un épanchement intra péritonéal de faible abondance finement échogène. L'exploration chirurgicale avait mis en évidence la présence de multiples masses d'allures tumorales au dépend du grêle (Figure 1) à développement exophytique dont 2 étaient surinfectées, et le geste avait consisté à faire des biopsies, lavage et drainage. Les suites post-opératoires ont été marquées après l'ablation des drains par l'issue du pus à travers les orifices de drainage. Ainsi vu l'aggravation du tableau clinique il a été référé pour une meilleure prise en charge. L'examen clinique trouvait un patient conscient stable sur le plan hémodynamique et respiratoire, apyrétique avec la présence d'une cicatrice médiane propre et 2 orifices de drainage: l'un au niveau du flanc droit et l'autre au flanc gauche, avec une sensibilité en regard de ces 2 orifices. Le reste de l'examen somatique était sans particularité. L'examen paraclinique indiquait une fonction rénale correcte, une hémoglobine à 10,3g/dl, normochrome normocytaire, les leucocytes à 13000/mm^3^, les plaquettes à 629000/mm^3^, la protéine C-réactive à 146 mg/l et le taux de prothrombine à 67%. Le scanner abdominal trouvait une volumineuse collection du flanc droit à contenu hydro-aérique et à paroi rehaussée après contraste, fistulisée à la peau avec la présence de quelques masses intéressant le jéjunum présentant un rehaussement périphérique après contraste avec une nécrose centrale, la plus grande mesurait 46x42mm (Figure 2). Ainsi nous avons conclu à une collection intra péritonéale au niveau du flanc droit fistulisée à la peau associée à de multiples masses grêliques évoquant en premier des GIST multiple. Un drainage radiologique de la collection intra-péritonéale fistulisée au niveau de la FID a été réalisé avec prélèvement du liquide pour étude cytobactériologique qui est revenu en faveur de bacilles gram négatif, ainsi le patient a été mis sous antibiothérapie adaptée. Une semaine après son hospitalisation le bilan biologique s'est amélioré avec un taux d'hémoglobine passé de 10,3 à 12g/dl, des leucocytes de 13000 à 11000/mm^3^ sans variation significative des autres paramètres. L'examen histologique des biopsies chirurgicales a rapporté une prolifération tumorale fusocellulaire dont l'aspect morphologique évoque des tumeurs stromales de type GIST. Nous avons retenu le diagnostic de tumeurs stromales de type GIST surinfectées. Une chimiothérapie a été instaurée après amélioration clinique et biologique du notre patient.

## Discussion

Les GIST sont les tumeurs conjonctives les plus fréquentes du tube digestif. La principale caractéristique de ces tumeurs est l'expression du marqueur CD117 (protéine kit ou c-kit). Seules les tumeurs c-kit positives sont considérées comme GIST sauf cas exceptionnels. Le diagnostic positif des tumeurs C-kit négatives peut être apporté par la recherche de mutations dans l'ADN tumorale des gènes Kit (qui code pour la protéine kit) et PDGFRA (qui code pour la chaîne A du récepteur du PDGF) [10]. Les GIST surviennent habituellement au niveau de l'estomac dans 40% à 70%, de l'intestin grêle de 20% à 40% et moins de 10% au niveau de l'œsophage, le côlon et le rectum [11, 12]. Les GIST sont uniques dans 95% des cas. Les localisations multiples sont très rares. Elles sont habituellement associées à des contextes particuliers comme les exceptionnelles formes familiales qui surviennent dès l'âge de 18 ans et sont caractérisées par la présence d'une mutation germinale de c-kit et PDGFRA. Sept familles ont été recensées dans la littérature comme porteuses de ce type de mutation [10]. Ce type de mutation constitutionnelle ne peut être écartée dans notre cas du fait qu'aucune recherche de mutation germinale n'a été réalisée, ni chez notre patient, ni dans sa famille.

La neurofibromatose de type 1 s'associe dans 7% des cas à des GIST multiples intéressant habituellement l'intestin grêle [10]. Notre patient ne présentait aucun des critères diagnostiques en faveur de la maladie de Recklinghausen. Les GIST affectent plus les hommes (55%) que les femmes avec un âge médian de 55-60 ans. La présentation clinique dans les cas de GIST est polymorphe et non spécifique. Elle dépend largement de la taille et de la localisation de la tumeur [13]. La présentation la plus fréquente est une douleur abdominale et / ou une hémorragie gastro-intestinale [14, 15]. Cela peut être aigu, avec méléna et hématémèse, ou sous forme de saignement insidieux chronique conduisant à l'anémie [15]. Notre cas concorde avec les données de la littérature où le patient était de sexe masculin âgé de 55 ans avec une anémie modérée normochrome normocytaire à 10,3g/dl mais cependant Cette anémie peut être aussi amputable aux pertes sanguines liées à l'intervention chirurgicale. Sept patients (7,6%) dans la série de Magdy A *et al.* avaient présenté une rupture et une péritonite [16]. Ils sont fréquemment diagnostiqués accidentellement pendant les études radiologiques ou les procédures endoscopiques ou chirurgicales [17]. Ce qui corrobore avec notre cas où la découverte a été accidentelle lors d'une intervention chirurgicale pour péritonite. Sur tomodensitométrie une GIST apparait comme une grande masse de tissus mous bien définie avec un rehaussement hétérogène. Les tumeurs sont généralement de densité variable et montrent un rehaussement inégal après le contraste intraveineux. Différents degrés de nécrose peuvent fréquemment être observés dans la masse [8, 18]. Les caractéristiques CT des GIST intermédiaires (5-10 cm) sont les suivantes: forme irrégulière, densité hétérogène et un modèle de croissance intraluminale et extraluminale [19].

Notre patient au scanner avait des masses au niveau du jéjunum avec un rehaussement périphérique et hétérogène après contraste avec une liquéfaction centrale (nécrose) pour la plus grande qui mesurait 46x42mm plus infiltration de la graisse mésentérique. Notre description scannographique corrélait parfaitement avec la littérature et a été l'élément capital pour évoquer le diagnostic de tumeurs stromales multifocales confirmé par l'anatomopathologie. Le consensus international (2002) a proposé de considérer toute GIST comme possédant un risque potentiel de malignité. La résection chirurgicale complète est le seul traitement potentiellement curatif des tumeurs stromales digestives [10]. Cependant, la multiplicité la répartition des lésions sur le grêle ainsi que la surinfection chez notre malade rendaient très difficile toute tentative de traitement radical. C'est dans ce contexte particulier que le traitement par l'imatinib prend toute son importance. Il s'agit d'un antagoniste pharmacologique de c-kit bloquant sa fonction de tyrosine kinase, indiqué dans les GIST avancées qu'elles soient non résécables, métastatiques ou récidivantes. Les données disponibles actuellement montrent que l'imatinib induit un taux de réponses objectives de 60 à 70%, avec 15 à 20% de maladies stabilisées et 10 à 15% de résistance primaire. Des résistances secondaires (échappement) sont désormais rapportées chez 10 à 30% des patients [10]. La particularité de notre cas est la découverte chez un individu dans les suites post opératoires d'une péritonite qui a été rarement rapporté et d'autre part le caractère multifocal trouvé au scanner.

## Conclusion

En somme il ressort de ce cas qu'en général que les tumeurs stromales gastro-intestinales n'ont pas de symptomatologie spécifique et qu'un tableau de péritonite chez une personne dans la soixantaine peut être leur mode de révélation. Le scanner permet d'évoquer le diagnostic.

## Conflits d’intérêts

Les auteurs ne déclarent aucun conflit d’intérêts.
